# Successful Management of Cesarean Scar Ectopic Pregnancy Using Local Methotrexate Injection and Balloon Catheter Compression: A Case Report

**DOI:** 10.7759/cureus.92400

**Published:** 2025-09-15

**Authors:** Natalie M George, Madhav Barot, Reham Haroun

**Affiliations:** 1 College of Osteopathic Medicine, Michigan State University, Detroit, USA; 2 Interventional Radiology, Henry Ford Health System, Warren, USA

**Keywords:** cesarean scar pregnancy, ectopic pregnancy, interventional radiology, methotrexate, obstetrics & gynecology (ob-gyn) services

## Abstract

Cesarean scar ectopic pregnancy (CSEP) is a form of non-tubal ectopic pregnancy. The diagnosis is made based on a combination of elevated beta human chorionic gonadotropin (β-hCG) levels and transvaginal ultrasound findings. In this case, a 32-year-old woman at 6.5 weeks gestation presented with abdominal cramping and vaginal bleeding. Transvaginal ultrasound confirmed a gestational sac implanted in the cesarean scar. After multidisciplinary consultation between obstetrics/gynecology and interventional radiology, the patient underwent ultrasound-guided intra-gestational methotrexate injection followed by balloon catheter compression. The results of the procedure included a rapid decline in β-hCG levels and resolution of the ectopic pregnancy. This case demonstrates the effectiveness of combining medical and mechanical interventions in early-stage CSEP and reinforces the importance of early diagnosis and coordinated care. Further research is needed to develop standardized treatment protocols for this rare condition.

## Introduction

Cesarean scar ectopic pregnancy (CSEP) is a rare and potentially life-threatening form of ectopic implantation that occurs within the myometrial scar of a previous cesarean delivery. In patients with a history of cesarean delivery, CSEP occurs in approximately one in 1,800 to one in 2,216 pregnancies and accounts for about 6% (one in 16.6) of all ectopic pregnancies [1]. Diagnosing CSEP can be challenging because patients may present with nonspecific symptoms such as abdominal pain, abnormal uterine bleeding, and elevated beta human chorionic gonadotropin (β-hCG) levels, a triad commonly seen in other ectopic pregnancies [2]. Transvaginal ultrasound is the primary diagnostic tool, but CSEP may resemble a cervical pregnancy or incomplete miscarriage on imaging [1]. As the rate of cesarean deliveries has increased by more than 50 percent over the past decade, the incidence of CSEP has also risen [3]. Although the condition is uncommon, it carries significant risks including uterine rupture and severe hemorrhage. Treatment has evolved from systemic intramuscular methotrexate administration to more localized and minimally invasive approaches, such as ultrasound-guided injection of methotrexate into the gestational sac [2]. This case highlights a successful multidisciplinary collaboration between interventional radiology and gynecology in treating CSEP and demonstrates the clinical importance of early diagnosis and coordinated management in improving outcomes.

## Case presentation

A 32-year-old G4P1021 woman at approximately 6.5 weeks gestation by last menstrual period presented to the emergency department with abdominal cramping and vaginal bleeding. She had no significant medical history but reported a prior planned low transverse cesarean section. A transvaginal ultrasound confirmed the presence of CSEP. Given the life-threatening nature of CSEP, treatment options were reviewed and the patient elected to undergo transvaginal ultrasound-guided intra-gestational methotrexate injection. Upon admission, both the obstetrics/gynecology and interventional radiology teams were consulted. Her initial β-hCG level was 257 mIU/mL (normal: <5 mIU/mL).

**Figure 1:**
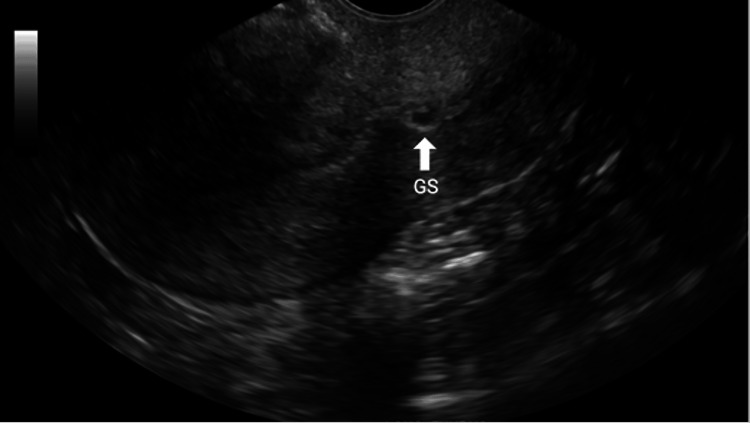
Transvaginal ultrasound showing a cesarean scar ectopic pregnancy Sagittal transvaginal ultrasound of the uterus demonstrates a 6.5-week gestational sac (GS) implanted within the anterior lower uterine segment at the site of a prior cesarean section scar. This finding is consistent with a cesarean scar ectopic pregnancy, characterized by implantation into the myometrium and fibrous scar tissue rather than the endometrial cavity.

**Figure 2:**
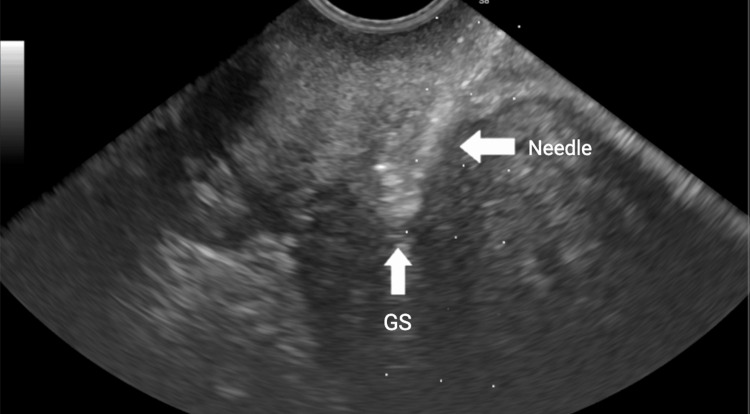
Transvaginal ultrasound showing a 22-gauge Chiba needle within the gestational sac Transvaginal ultrasound image demonstrates placement of a 22-gauge Chiba needle into the gestational sac (GS) under real-time guidance. The arrow identifies the needle within the sac following methotrexate injection.

**Figure 3:**
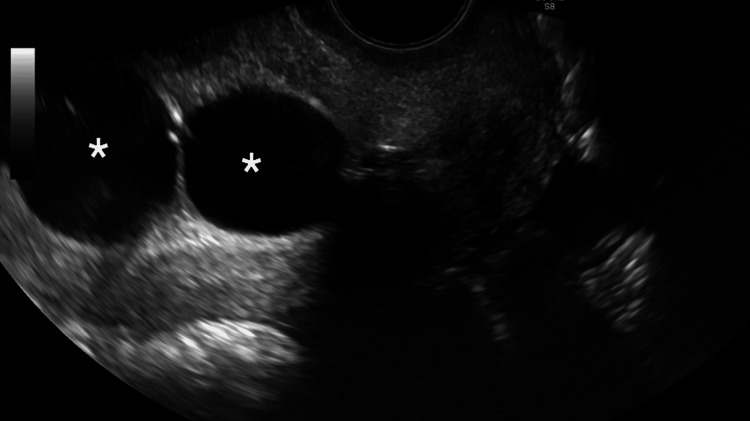
Transvaginal ultrasound showing inflated double-balloon catheter after methotrexate injection Transvaginal ultrasound demonstrates an inflated double-balloon catheter following methotrexate injection for treatment of a cesarean scar ectopic pregnancy. Asterisks mark the anechoic regions corresponding to the inflated upper (uterine) and lower (vaginal) balloons, which exert compression on the gestational sac located within the cesarean section scar site.

**Figure 4:**
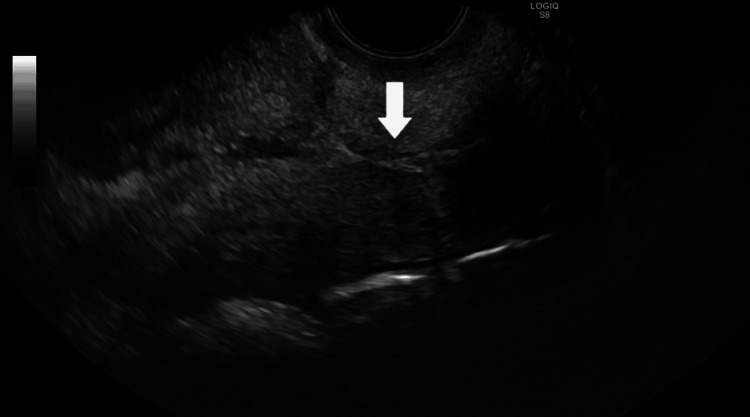
Postoperative day 2 transvaginal ultrasound showing resolution of the gestational sac Postoperative day 2 transvaginal ultrasound demonstrates the prior low transverse cesarean section scar with no sonographic evidence of a gestational sac. The white arrow indicates the absence of a gestational sac within the endometrium, consistent with successful treatment of the cesarean scar ectopic pregnancy.

In the ultrasound suite, the patient was sedated and placed in the lithotomy position. A preliminary transvaginal ultrasound confirmed a gestational sac with cardiac activity located within the cesarean scar (Figure 1). Under transvaginal sonographic guidance, the interventional radiologist advanced a 22-gauge Chiba needle into the gestational sac, and 25 mg of methotrexate was injected by the gynecologist (Figure 2). An immediate post-procedure ultrasound confirmed cessation of cardiac activity. Following the injection, a double-balloon catheter was inserted under ultrasound guidance. The uterine balloon was inflated with 10 mL of saline, followed by inflation of the vaginal balloon with 10 mL of saline beneath the gestational sac (Figure 3). Complete collapse of the gestational sac was confirmed using ultrasound (Figure 4). Hemostasis was achieved prior to removal of all instruments and the patient tolerated the procedure well with minimal blood loss estimated at less than 50 mL. The patient was discharged with instructions to return in two days for balloon catheter removal and serial β-hCG monitoring. On postoperative day two, the catheter was successfully removed, and her β-hCG level had decreased to 25 mIU/mL.

## Discussion

Ectopic pregnancies are categorized as tubal or non-tubal. CSEP is a rare form of non-tubal ectopic pregnancy [4]. The incidence of CSEP has increased alongside the rising number of cesarean deliveries, which have doubled since 2000 [5]. The diagnosis of CSEP requires both an elevated β-hCG level and specific findings on transvaginal ultrasound. These findings typically include an empty uterine cavity with a clearly visualized endometrium, an empty cervical canal, a gestational sac embedded in the anterior portion of the lower uterine segment corresponding to the cesarean scar, and a thin or absent layer of myometrium between the sac and the bladder [5]. Early detection and timely termination of the pregnancy are critical to reducing morbidity and mortality. If left untreated, CSEP poses serious risks including severe hemorrhage, uterine rupture, placenta accreta spectrum disorders, hysterectomy, and maternal death [6,7].

Multiple treatment options are available for managing CSEP, although no single gold standard has been universally accepted. Current treatment algorithms are based primarily on the patient’s hemodynamic stability. Hemodynamically stable patients may be treated with medical therapy, surgical intervention, or expectant management, while unstable patients require immediate surgical treatment [8]. The literature describes a variety of treatment modalities, including systemic methotrexate (MTX), local MTX injection, suction curettage, balloon catheter compression, high-intensity focused ultrasound, interventional radiology procedures, and hysteroscopy [8]. These options can be used alone or in combination, as demonstrated in the case presented in this report.

Among medical therapies, systemic intramuscular MTX has been shown to have lower success rates and often necessitates additional interventions to terminate the pregnancy. In contrast, local injection of MTX into the gestational sac has demonstrated higher efficacy [8]. For early gestational CSEP cases, balloon tamponade or hysteroscopy has also been found to be effective [8]. As there is no established protocol for the management of CSEP in this setting, the interventional radiology team, in collaboration with the obstetrics and gynecology service, elected to proceed with a combined medical and mechanical approach to ensure termination of the pregnancy. This strategy was favored by the treating physicians and highlights the role of multidisciplinary decision-making to promote optimal outcomes in the absence of a standardized protocol. Post-procedure, the patient was monitored with serial β-hCG levels and observed for complications such as uterine or cervical perforation and infection.

## Conclusions

CSEP is a rare but serious condition with a rising incidence linked to increased cesarean deliveries. Early diagnosis through transvaginal ultrasound and β-hCG monitoring is critical for timely management. In this case, a multidisciplinary approach led to a successful treatment using local methotrexate injection and balloon catheter compression at 6.5 weeks of gestation. However, further research is needed to establish a standardized treatment algorithm, as no universally accepted gold standard currently exists for CSEP management.
